# Health and safety matters! Associations between organizational practices and personal support workers’ life and work stress in Ontario, Canada

**DOI:** 10.1186/s12913-017-2355-4

**Published:** 2017-06-21

**Authors:** Isik U. Zeytinoglu, Margaret Denton, Catherine Brookman, Sharon Davies, Firat K. Sayin

**Affiliations:** 1 DeGroote School of Business, McMaster University, Hamilton, ON Canada; 2 Department of Health, Aging & Society and Gilbrea Centre, McMaster University, Hamilton, ON Canada; 3 Catherine Brookman Consulting & Associates, Hamilton, ON Canada; 4 McMaster University, Hamilton, ON Canada

**Keywords:** Personal support workers, Life and work stress, Organizational practices, Support at work, Preferred hours

## Abstract

**Background:**

The home and community care sector is one of the fastest growing sectors globally and most prominently in mature industrialized countries. Personal support workers (PSWs) are the largest occupational group in the sector. This paper focuses on the emotional health of PSWs working in the home and community care sector in Ontario, Canada. The purpose of this paper is to present evidence on the associations between PSWs’ life and work stress and organizational practices of full-time and guaranteed hours, and PSWs’ perceptions of support at work and preference for hours.

**Methods:**

Data come from our 2015 survey of 1543 PSWs. Dependent variables are life and work stress. Independent variables are: objective organizational practices of full-time and guaranteed hours, and subjective organizational practices of perceived support at work, and preferred hours of work. Descriptive statistics, correlations and ordinary least square regression analyses with collinearity tests are conducted.

**Results:**

Organizational practices of employing PSWs in full-time or guaranteed hours are not associated with their life and work stress. However, those who perceive support from their organizations are also the ones reporting lower life and work stress. In addition, those PSWs perceiving support from their supervisor report lower work stress. PSWs would like to work in their preferred hours, and those who prefer to work more hours report lower life and work stress, and conversely, those who prefer to work less hours report life and work stress.

**Conclusion:**

For PSWs in home and community care, perceived support from their organizations and supervisors, and employment in preferred hours are important factors related to their life and work stress.

## Background

Home and community care sector is one of the fastest growing sectors globally and most prominently in mature industrialized countries [1–3]. With the population aging, the preference to stay in one’s own home, and ongoing shortages of care workers [3–7], has resulted in an increased demand for home and community care services particularly for the care services provided by personal support workers (PSWs) [8, 9]. There is similarly a high need for elder care in nursing homes in mature industrialized countries [10, 11]. In addition, health care sector reforms in the past few decades have resulted in reduced budgets for home and community care making it difficult for organizations to hire sufficient number of workers to provide services for increased demand [1–6, 12, 13].

This paper focuses on home and community care services in Ontario, Canada. Home and community care services in Canada help people receive care at home or in a retirement home, and does not refer to care provided in a hospital or long-term facility such as a nursing home [14]. Home and community care services are delivered by regulated health care professionals, such as nurses and therapists, non-regulated workers such as personal support workers, and unpaid workers such as volunteers, and family and friends of care recipients [14]. In Canada, home and community care services are mainly delivered by provincial, territorial and some municipal governments, though individuals can purchase these services privately. The federal government also provides home and community care services to First-Nations on reserve and Inuit in designated communities, among others [14]. These services are funded through transfer payments from the federal Government to provinces, territories and some municipalities. In Ontario, where this study took place, Community Care Access Centres (CCAC) are the organizations that receive funding and distribute it to service organizations through a competitive bidding process. Personal support workers work for these service organizations or through organizations directly funded by the Local Health Integrated Networks (LHINS) The employing organizations can be private for-profit, private not-for-profit or public not-for-profit.

There is no universally accepted terminology for the PSW occupation. For example, they are called health support workers in the U.K. [15], home care workers [16, 17] or home health care aides in the U.S. [18, 19], home health care workers or aides in Europe [7], home care workers in Japan [3] and home care attendants in Taiwan [20]. In Canada, they used to be called home support workers [1] or visiting homemakers [21] though now they are more commonly referred to as personal support workers [12, 22, 23]. The PSW occupation is non-regulated in Canada, meaning that a licence from a regulatory body is not required to be employed in the sector [14].

Home and community care PSWs provide care to patients discharged from hospitals, the elderly including those with dementia, and persons with disabilities. The workplace of the home and community care PSWs is the home of the person receiving care. PSWs perform a variety of tasks each tailored to the needs of the person receiving care. Some of the tasks performed are: (1) activities of daily living – personal care (bathing, feeding, dressing, toileting), transferring (from sit to stand, walking), light housekeeping, and child care; (2) instrumental activities of daily living – menu planning, shopping, meal preparation, providing transportation or accompanying clients, educational and recreational assistance; (3) clinical care services – measuring a client’s blood pressure, taking temperature, pulse, specimens; and (4) delegated tasks – administration of suppositories, colonic irrigation, enemas (bowel disimpaction), or medication; maintaining inventories; and supervising exercise routines [4, 12, 22].

### Purpose and contribution of the study

The purpose of this paper is to present evidence on the associations between PSWs life and work stress and organizational practices, perceptions of support at work and preference for hours of work. Research shows that PSWs experience stress due to the nature of their work and work environments in Canada and elsewhere including U.S., UK, Europe, and Japan [3, 7, 11–13, 15–17, 21, 24]. Similarly, nurses, nursing aides, nursing assistants and nursing health care staff employed in elderly care in long-term care facilities/ nursing homes in Canada [25], Japan [26], Taiwan [20] Switzerland [27], Finland [28], U.S. [29] and UK [30] report stress at work. The stress in health care environments is so unique to the sector that it is sometimes referred as a ‘stress of conscience’ where health care staff experience a troubled conscience because they would like to provide the best quality care but are unable to do so [31] for a variety of reasons. This paper contributes to academic and practitioner knowledge by showing the relationship between life and work stress and organizational practices, worker perceptions of support at work, and preferences for hours of work of home and community care PSWs.

### Theoretical foundation, empirical knowledge and hypotheses

Our research is informed by the stress coping theory of Lazarus [32] and job stress theory of Ironson [33] in developing the conceptual model and in introducing organizational practices, perceptions and preferences to be tested in the model. The experience of life and work stress is an individual experience that can vary from one person to another. According to Lazarus’s transactional theory [32], when an individual is faced with a potentially stressful situation, s/he evaluates it as a threat or challenge, and depending on their coping abilities and resources, some are able to cope with stress, and others show strain. Focusing on work stress, the demand-control model of Karasek [34] states that when workers have high demands and low control at work they present symptoms of stress. PSW work is a high demand–low control work, where workers are expected to provide care in the allocated time period. The care, i.e. tasks, are usually assessed and decided upon jointly with the client and case managers for each client, while PSW supervisors oversee the scheduled work for each PSW and inform them of the clients to visit for care giving, tasks to be performed for each client, and time allocated for each task.

**Fig. 1:**
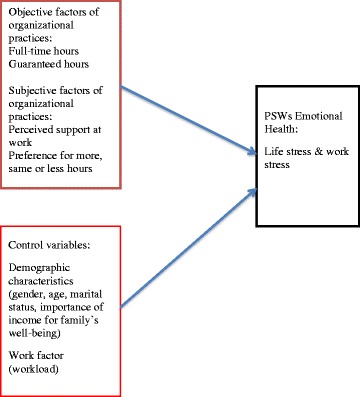
The conceptual model of organizational practices associated with PSWs life and work stress

Empirical research has shown a number of demographic characteristics and work factors that can be associated with stress. As we present in Fig. 1, we control for the possible associations of some of those factors with stress. For demographic characteristics of gender and marital status, particularly for women, some studies find association with stress [35, 36] and others do not. For example, our studies with PSWs show no association with gender and marital status and various health outcomes arguing that it is the work environment factors, not individual characteristics that are related to stress [21, 24]. Age does not seem to have a clear association with stress in some studies [36] though in our studies with PSWs those who are older report lower stress [21, 24]. We also include the importance of income for the family’s economic well-being as a factor to be controlled in this study. PSWs are the lowest paid workers in the health care labour force in US, and financial worries can be a source of stress [37]. In our studies of home care workers and nurses, the importance of income from the PSW work for their family’s well being showed to be associated with work attitudes and behaviours and/or health [24, 38, 39]. For nurses in Ontario, Canada research showed that as the importance of income for their family’s well-being increased so did their stress level [39]. The work characteristic of workload is shown to be associated with the work stress of health care workers [10, 20, 27–29, 31, 36, 38, 40, 41] and this factor is included as a control variable in our study.

As presented in Fig. 1, organizational practices can be observed as objective factors of hours of work (full-time hours or not), and whether hours are guaranteed or not. Organizational practices as seen through the lens of workers are subjective factors that can affect workers perceptions and can match or mismatch with their preferences. Subjective factors in our study are perceived support at work and preferences for hours We test the relationships of these objective and subjective factors to life and work stress .

An empirical study on the hours of work with geriatric care workers in Germany shows that as the amount of working hours per week increased there was an increase in stress and strain among workers [11]. However, for those working part-time hours, there was low stress [11]. Similarly in US, a study of female nursing home assistants showed that the odds of adverse mental health increased with increased hours of work [42]. Thus, we hypothesize that:

Hypothesis 1. Working full-time hours will be positively associated with PSWs life and work stress.

Many organizations in home and community care in Ontario, Canada hire PSWs as needed and their work hours are not guaranteed [4, 12, 13]. The organizational practice of guaranteed hours of work is security for worker, because guaranteed hours provide continuity in pay, benefits and financial security [24]. Research from other countries in similar occupations or in the health care sector show the importance of guaranteed hours for the emotional health of workers. A study from Finland with registered nurses has found insecure work contracts to be associated with reduced well-being indicators such as psychological distress [43]. In another study, nurses’ perception of good work in Sweden included work security and a steady income [44]. A study in Germany also showed that predictability in hours and decreased job insecurity were important in lowering geriatric care workers stress [11]. Guaranteed hours of work can provide security to PSWs; and those who work in guaranteed hours might also be the ones reporting lower life and work stress.

Hypothesis 2. Guaranteed hours will be negatively associated with PSWs life and work stress.

A systematic review of working in nursing homes found low social support at work in smaller-scale nursing homes to affect the quality of working life [10]. Studies found support at work to be associated with lower levels of stress among nursing home workers, home care workers and nurses in Australia, Canada, Sweden and Germany [8, 10–12, 21, 24, 31, 40] though a study from US on nursing aide workers found effects of managerial and co-worker support on stress to be minor [29]. Another study from Sweden on nurses’ perceptions of good work showed having pleasant and appreciative fellow workers, i.e. co-worker support, and having a fair and understanding manager, i.e. supervisor support, as important factors at work [44]. Based on these studies we hypothesize that:

Hypothesis 3. Support at work (organizational, supervisor, and/or peer support) will be negatively associated with PSWs life and work stress.

Working in preferred employment conditions can be associated with workers’ reporting low levels of stress. Our study of nurses employed in hospitals in Ontario, Canada found that it is important for these workers to be employed in their preferred hours, whether it is more, the same or less hours to retain them in the workplace [39]. Another study showed that elderly care nursing staff preferred to control their work schedules and this was related to lower strain [41]. Though our study is not on the control over work hours, we can infer from this [41] and the preferred hours study [39] that PSWs in our study would also like to work in their preferred hours. We would also argue that those who prefer to work more hours would also be the ones reporting lower life and work stress, and those preferring to work less hours would be the ones reporting higher life and work stress.

Hypothesis 4. Preference to work more hours will be negatively associated with PSWs life and work stress.

## Methods

### Research design

This paper is based on our Ontario-wide 2015 survey data collected under the ‘*The PSW Health and Safety Matters!*’ title [hereafter the survey] (http://www.pswshaveasay.ca). The project is guided by a Research Advisory Committee (RAC) which includes the representatives from two home care organizations’ associations (employer associations), two of the largest unions in the sector, a PSW employee association, a health and safety association with expertise on the occupational health of the home and community based PSWs, and the principal investigator, co-lead, and three co-investigators of the project (see acknowledgment section).

### Population, sample and data collection process

The study population is composed of all PSWs employed in the home and community care sector in Ontario, Canada (estimated 26,000^1^ [45, 46]). A total of 2341 PSWs responded to the vey. This study uses data from respondents who completed the entire survey (i.e., came to the last page of the online survey) and selected ‘submit the survey’ option, and for the print-mail survey, mailed it back to us (*n* = 1746). In the analyses, observations with missing values are excluded and, thus, the sample size used in this paper is 1543. Using the sample size formula [47] for a population of 26,000, with 95% confidence interval and 3% margin of error we needed 1026 respondents. Our sample size for this paper well exceeds the sample size needed to accurately reflect the population (with 95% confidence interval) and the true value the population would give to our questions (with 3% margin of error). In addition, for conducting multiple regression analyses with small effect size, our data is more than sufficient [48].

After receiving ethics approval from McMaster University Research Ethics Board (MREB-2014-132) the survey process started. The pilot testing of the understanding and completion of the survey in both an on-line format and print-mail format was conducted with 10 PSWs from St. Clair West Services for Seniors, a community services home care organization. The pilot testing did not show any issues with the survey in either format.

In collecting data, the research team used three approaches. First, we attempted to reach as many PSWs as possible by sending out email blasts by partner organizations that are members of the Research Advisory Committee; Ottawa West Community Support; and the PSW Registry. These email blasts encouraged PSWs to participate in our survey by going to the study website (http://www.pswshaveasay.ca). The project website displayed a video which explained the survey. Reminder email blasts were also sent to encourage participation. A second approach for recruitment was through the placement of advertisements on several organizations’ websites and a project article distributed in many organizational newsletters to promote our survey. Some organizations also promoted our survey through Twitter. A third strategy to recruit survey participants was to incorporate strategies at the organization level. We developed several tools for organizations to use to encourage their employees to complete our survey: a flyer was provided promoting our survey which could be sent out to employees; a “newsletter article” that organizations could include in their monthly newsletters was provided; and a mini Health and Safety In-service PowerPoint presentation which incorporated our survey was developed. Organizations were invited to use these strategies to promote participation in our survey.

PSWs were asked to respond to the survey only if they were a community-based PSW. The on-line survey was collected using LimeSurvey, an on-line web application. Most responses were to the on-line survey, although some preferred print-mail surveys. Both on-line and print-mail surveys started with confirmation of consenting to participate in the survey. Although respondents were encouraged to complete the entire survey, they were not required to do so and respondents were not required to answer every question in the survey. Survey respondents were given minor incentives to complete the survey. On-line survey respondents were given an option to enter a draw to win one of several gift cards upon completion of the survey and print-mail survey participants were given a very small (amount) gift card that was mailed along with the survey.

### Instrument and measures

The instrument of the study was the self-completion of *The PSW Health and Safety Matters Survey* put together by the research team using their own questions, and theirs and others’ published scales [49]. Unless specifically explained below, all variables were measured on a five-point Likert scale anchored with ‘1 = strongly disagree’ to ‘5 = strongly agree’. The equal interval assumption is used for the Likert scale measurement of dependent, independent and control variables. To create scores for each scale, responses to each item were summed together, with some items reverse coded as suggested by the scale developer. In constructing the scales, in order to reduce missing data, if missing values for each item in a scale were less than 5 %, we replaced the missing values in the item with the mean of the item. As referenced below, all scales used in this paper have been previously developed and validated in earlier studies.

**Dependent variables** are life and work stress. **Life stress** is worded as ‘thinking about the amount of stress in your life, would you say that most days are…?’ and **work stress** is worded as ‘working as a PSW in the community, in the past twelve months, would you say most days at work were…?’ Responses are coded on a five-point Likert scale with ‘1 = not at all stressful’ to ‘5 = extremely stressful’. These questions are from the Canadian Community Health Survey (CCHS) [50].

Organizational practices are **independent variables**. Objective measures of organizational practices are providing full-time hours and guaranteed hours. The **full-time hours** variable is from the question ‘approximately how many hours do you work per week as a PSW in the community?’ with those answering 30 h or more coded as full-time hours, using an approach similar to Statistics Canada’s calculation of full-time versus part-time hours. **The guaranteed hours** variable is from the question ‘are your hours guaranteed?’ with response as a dummy variable, coded 1 = yes, 0 = no. Subjective measures of organizational practice are support at work and preference for hours. **Support at work** is examined as perceived organizational, supervisor and peer support. Organizational support and supervisor support are, six-item and seven-item scales, based on Denton et al. [21] and Zeytinoglu et al. [38] with two questions included from our survey [49]. Peer support is from Denton et al. [21] with four items. A sample item for each scale is ‘your organization supports you in times of personal crisis, illness or needing time off to help care for other family members’, ‘you have the opportunity to talk openly with your supervisor about work-related problems’ and ‘your co-workers are helpful in getting the job done.’ Measurements of scales are as explained above. The Cronbach’s αs for scales indicate high internal reliability (See Table 1 along the diagonal). **Preference for more, same or less hours** is based on the question ‘would you prefer to work more or less hours per week as a PSW in the community?’ with response items of more, same or less hours per week (each as a dummy variable, coded 1 = yes, 0 = else).

**Table 1:** Descriptive statistics and correlations between life stress and work stress (dependent variables) and organizational practices, perceptions and preferences (independent variables)

Variables	M (SD)	1	2	3	4	5	6	7	8	9	10
1. Life stress	2.908 (.860)	-									
2. Work stress	2.954 (.912)	**0.507**	-								
3. Full-time hours	.593 (.491)	**−**0.001	**0.058**	-							
4. Guaranteed hours	.406 (.491)	**−**0.018	**−**0.021	**0.190**	-						
5. Organizational support	20.823 (4.570)	−**0.242**	−**0.306**	0.025	**0.070**	*0.81*					
6. Supervisor support	24.859 (6.700)	−**0.174**	−**0.263**	0.024	**0.065**	**0.710**	*0.94*				
7. Peer Support	13.645 (3.434)	−**0.113**	−**0.100**	**0.089**	**0.146**	**0.428**	**0.457**	*0.85*			
8. Prefer more hours	.509 (.500)	**−**0.046	−**0.055**	−**0.269**	−**0.245**	**−**0.008	**−**0.026	−**0.073**			
9. Prefer same hours	.415 (.493)	**−**0.036	**−**0.035	**0.199**	**0.226**	**0.084**	**0.077**	**0.108**	−**0.858**		
10. Prefer less hours	.0758 (.265)	**0.153**	**0.170**	**0.138**	0.043	−**0.142**	−**0.094**	−**0.063**	−**0.292**	−**0.241**	

Note: Cronbach’s α values are presented in italics along the diagonal. Correlation coefficients at the .05 or lower level of significance are in bold. *n* = 1543

### Control variables

**Demographic characteristics** are gender, age, marital status, and importance of income for family’s economic wellbeing. Gender is a binary variable (female = 1, male = 0), with females as the majority of workers in the sample (93%). Age is coded as number of years with respondents’ average age being 49. Marital status is measured as 1 = married/common law relationship, 0 = other, with 66% married/common law. Importance of income for family’s economic wellbeing is measured on a five-point Likert scale as explained above; with this issue being very important for respondents (*M* = 4.56, *SD* = 0.75). The percentage distribution of gender, age and marital status are similar to those reported in earlier studies [1, 12, 21]. For the **work factor**, workload is a scale including both physical and psychosocial work environment items [21, 24]; a sample item is “you have too much to do on the job.” The scale has high internal reliability (*α* = .89) and results show the workload is heavy for PSWs (*M* = 22.08, *SD* = 5.76, scale range: 7 to 35).

### Analysis

Descriptive statistics, correlations and Ordinary Least Squares (OLS) regressions are conducted. Descriptive statistics and correlations are presented in Table 1, and OLS regression results are presented in Table 2. The subjectively assessed variables may not be completely independent from each other, and thus collinearity diagnostics (tolerance and variance inflation factor analyses) were also conducted. Collinearity with dependent variables was not found. STATA 14 is used in the analysis.

**Table 2:** Organizational practices, perceived support at work, and preference for work hours associated with personal support workers’ life stress and work stress (OLS regressions)

Variables	Life stress	Life stress	Work stress	Work stress
	B (SE)	β	B(SE)	β
Constant	3.017 (.207)^c^	N/A	2.134 (.197)^c^	N/A
*Objective organizational practices:*				
Full-time hours	−.077 (.045)	−.044	−.005 (.043)	−.003
Guaranteed hours	.022 (.044)	−.012	−.037 (.042)	−.020
*Subjective organizational practices:*				
Organizational support	−.028 (.007)^c^	−.147	−.021 (.006)^c^	−.103
Supervisor support	.001 (.005)	−.009	−.011 (.004)^b^	−.082
Peer support	−.008 (.007)	−.033	.005 (.007)	.020
Preference for more hours	−.105 (.046)^a^	−.061	−.108 (.044)^a^	−.059
Preference for same hours	Reference	Reference	Reference	Reference
Preference for less hours	.295 (.082)^c^	.091	.242 (.079)^b^	.070
*Control variables:*				
Gender	.059 (.083)	.017	.019 (.079)	.005
Age	−.006 (.002)^c^	−.078	−.003 (.002)	−.042
Marital status	.077 (.044)	.042	.031 (.042)	.016
Importance of income for family’s economic wellbeing	.180 (.046)^c^	.097	.172 (.044)^c^	.087
Workload	.032 (.004)^c^	.212	.069 (.004)^c^	.434
Adj.R^2^	.13		.30	
*n*	1543		1543	

^a^Statistically significant at the .05 level; ^b^ at the .01 level; ^c^ at the .001 level

## Results

### Descriptive statistics

As presented in Table 1, PSWs report that most days in their lives and at work were a bit stressful in the past 12 months. Working full-time hours is more prominent among the PSWs, however, 41% work part-time hours. A large percentage of PSWs work with no guaranteed hours and only 41% report having guaranteed hours. PSWs strongly agree that their organization, their supervisors, and co-workers support them at work. Half prefer to work more hours, close to the other half prefer the same hours of work, and only 7% prefer to work less hours.

### Correlations

Table 1 shows correlations between dependent and independent variables. Life stress is significantly and positively correlated with work stress, and we note that the correlation is particularly high. Correlations between life stress and independent variables show that organizational, supervisor and peer support are significantly and negatively, and preference for less hours is significantly and positively correlated with life stress. Work stress shows the same pattern with the additional factors of working full-time hours showing significant and positive correlation and preference for more hours showing significant and negative correlation. Correlations between dependent, independent and control variables are also conducted (results are available from the first author).

### Regressions

As presented in Table 2, we test *Hypotheses 1–4* for each dependent variable, controlling for possible associations of demographic and work factors. Starting with the second column titled *life stress*, as hypothesized, organizational support (*Hypothesis 3*) and preference for more hours (*Hypothesis 4)* are negatively and significantly, and preference for less hours is positively and significantly associated with life stress. Other hypothesized associations are not significant. The magnitude of standardized coefficients (βs), presented in third column titled *life stress,* show that after workload, organizational support is important for PSWs, and importance of earnings for family’s economic well-being, and preference for more hours are important factors associated with life stress. The variables in the model explain 13% of variance (Adj.R^2^ = 0.13).

In column 4 titled *work stress*, the regression analysis shows that, as hypothesized, organizational and supervisor support (*Hypothesis 3*) and preference for more hours (*Hypothesis 4)* are negatively and significantly, and preference for less hours is positively and significantly associated with work stress. Other hypothesized associations are not significant. The magnitude of standardized coefficients (βs), in column 5, show that after workload, organizational support is important for PSWs, and after importance of earnings for family’s economic well-being, supervisor support, preference for less hours, and preference for more hours are important factors associated with work stress. The variables in the model explain 30% of work stress for PSWs (Adj.R^2^ = 0.30).

## Discussion

### Main findings

With respect to life stress, as hypothesized, PSWs perceiving organizational support and preferring to work more hours are also the ones reporting less stress in their lives, and those preferring to work less hours are also the ones reporting life stress. Focusing on work stress, we find that, as hypothesized, PSWs perceiving support from their organization and supervisor are also the ones reporting less work stress. This finding supports earlier studies in nursing [10] focusing on nursing home workers, home care workers and nurses in Australia, Canada, Sweden and Germany [8, 10–12, 21, 24, 31, 40, 44]. In addition, as hypothesized, PSWs preferring more hours report low levels of work stress, and conversely, those who prefer less hours report higher levels of work stress. This finding is in line with earlier studies on nurses preferences in hours and retention [39]. The results of this study also supports an earlier study that showed control over work schedules to be related to lower strain [41]. However, contrary to our hypotheses, neither the full-time hours nor the guaranteed hours are related to life and work stress reported by the PSWs in our study. These findings do not support earlier studies on related occupations of nurses, nursing home aides and home care workers in other countries [11, 24, 42–44].

### Implications

Whether working full-time or part-time, or having guaranteed hours or not, are not related to PSWs life and work stress. It is their perceptions of support at work and preference for working more or less hours that is related to their life and work stress, similar to findings in related health care occupations in other countries [8, 10–12, 21, 22, 24, 40, 41]. We know that community based organizations typically have a high PSW to supervisor ratio which is both a result of funding and organizational structures [12]. With the ongoing challenge of limited funding for other than direct client service (e.g. very little administrative/planning funding), decision-makers have the opportunity to create and test out different models of PSW worker support [51]. In some home and community care organizations PSW Team Leads are being used to support other PSWs for particular tasks and training [52]. With more clients needing medication management, decision-makers need to find models of support in order to grow PSW expertise and assist in their experience of working in healthier work environments, particularly in a less stressful work environment, which can, in turn, have a spillover effect on life stress. Recognizing that not all PSWs will receive the training necessary to deliver this more complex type of care nor feel comfortable with this type of care, there exists an opportunity for decision-makers to work with PSWs to develop new structures for peer and supervisory support beyond the existing status quo of PSW to supervisor ratio. Perceptions of such support at work, whether it comes from the organization or the supervisor, could be related to PSWs reporting lower life and work stress. In addition, Ontario government’s recent budget commitment to partnering with labour unions and employers to support PSW training can address the workers concerns of the delivery of complex care and can be associated with lower stress levels among PSWs [53].

The study showed that PSWs preference for hours of work, whether more or less, is related to their emotional health. This would denote an opportunity for decision-makers to consider co-ordinating the PSW workforce based on desired number of work hours. The association of the hours with life and work stress lends itself to a healthier workforce by catering to the preferences of each worker. This can take more administrative time but the end result of a healthier workforce is, in our view, worth the managerial time.

### Strengths, limitations, and suggestions for future research

This study has a number of strengths and limitations and future research directions derived from the results. Our study is the first province-wide data collection of occupational health and safety of PSWs in Ontario. The PSWs are a large occupational group not only in Ontario but also in Canada, and there is no data that is available to analyze the relationship between their working conditions and health [54]. The strength of our data is the number of respondents. The sample size is statistically representative of Ontario’s home and community care PSWs, and the sample size is sufficiently large to allow us to do the analyses with many dependent and independent variables and controlling for many other factors [47, 48]. There is, however, a limitation, and that is, there is no PSW-specific data collected by the government’s health and safety agency, Workplace Safety and Insurance Board (WSIB) of Ontario to compare the work and life stress reported by our respondents. We recommend WSIB to be more specific in its data collection on this significant and increasing number of workers in Ontario and collect data on emotional and physical health of PSWs. Perhaps a national level-data collection, through Statistics Canada, on PSWs’ work, health and safety can be considered as well. Another limitation of our study is that we were not able to collect organization-specific data and match individual respondents with data from their employers. We recommend future studies to build on our study and extend it by collecting a matched employer-employee data.

## Conclusion

This study provides a comprehensive analysis of many factors that are related with PSWs emotional health of life and work stress. The study shows that organizational practices and how they are perceived and preferred by the PSWs are related to reporting good or poor emotional health. Particularly, our study indicates the importance of perceived support at work and working in preferred hours to be related to reporting positive health. It appears that it is not hours of work or having guaranteed hours, but PSWs perception of support at work and preferences for work hours that are related to their life and work stress. Based on findings we recommend that decision-makers pay particular attention to each individual PSWs perceptions and preferences in order to provide healthy and safe work environments for these workers. Perhaps decision-makers could use these findings as an opportunity to work together with PSWs to develop new models for client care delivery and scheduling of hours as well as developing new infrastructure models for improved supervisory and/or peer support. Our findings make important contributions to both academic and practitioner knowledge, highlighting the importance of support at work and employment in preferred hours associated with workers reporting of positive emotional health outcomes.

## Abbreviations

CCHS: Canadian Community Health Survey
CUPE: Canadian Union of Public Employees
OCSA: Ontario Community Support Association
OHCA: Ontario Home Care Association (Home Care Ontario)
OPSWA: Ontario Personal Support Worker Association
PSHSA: Public Services Health & Safety Association
PSNO: Personal Support Network of Ontario
PSWs: Personal Support Workers
SEIU: Service Employees International Union
the survey: *The PSW Health and Safety Matters Survey*
WSIB: Workplace Safety and Insurance Board

## Footnotes

1 This number is a composite derived from information collected from a variety of sources, including reports on long-term care facilities, employer and association websites and relevant studies. Three factors may affect its accuracy. 1) The number of PSWs hired privately, making total employment potentially higher. 2) Some individuals who perform the same core tasks as many PSWs may work under other job titles and, therefore, not identify with the occupational group. This would reduce the reported size of the workforce. 3) Some employers record full-time equivalent (FTE) positions rather than full-time staff. This potentially inflates their ‘head-count’ where, for example, a single PSW could pick up double shifts for one or more employers.
